# 

**Published:** 1873-11

**Authors:** 


### Books and Pamphlets Received.

A Treatise on the Diseases of the Eye. By J. Soelberg Wells, F. R. C. S., etc. Second American from the Third English Editions with additions. Philadelphia: Henry C. Lea, 1873. Buffalo: T. Butler & Son.

Lectures on Clinical Medicine. By A. Trousseau, late Professor of Clinical Medicine in the Faculty of Medicine, Paris. Translated from the Third Revised and Enlarged Edition. By Sir John Rose Cormack, M. D., F. R. S. E, and P. Victor Bazire, M. D., Complete in two Volumes. Philadelphia: Lindsay and Blakiston, 1873. Buffalo: T. Butler & Son.

Lacerations of the Female Perineum; and Vesico Vaginal Fistula; Their History and Treatment. By D. Hayes Agnew, M. D. Philadelphia: Lindsay and Blakiston, 1873. Buffalo: T. Butler & Son.

Report on the Diseases of Indiana for the year 1872 By Geo. Sutton, M. D., Chairman of the Committee.

Lectures on Diseases and Injuries of the Ear. Delivered at St. George's Hospital. By W. B. Dalby, F. R. C. S., M. B., Cantab. With Illustrations. Philadelphia: Lindsay & Blakiston, 1873. Buffalo: T. Butler & Son.

An Introduction to Practical Chemistry, including Analysis. By John E. Bowman, F. C. S. Edited by Charles L. Bloxham, F. C. S. Philadelphia: Henry C. Lea, 1873. Buffalo: T. Butler & Son.

The Transactions of the American Medical Association, Vol XXIV. Philadelphia: printed for the Association.

On the Mechanical Treatment of Disease of the Hip-Joint. By Charles Fayette Taylor, M. D. New York: Wm. Wood & Co., 1873. Buffalo: H. H. Otis.

A Practical Treatise on Diseases of the Ear, including the Anatomy of the Organ. By D. B. St. John Roosa, M. A., M. D. With Illustrations. New York: Wm. Wood & Co., 1873. Buffalo: H. H. Otis.

The Physician's Visiting List for 1874. Philadelphia: Lindsay & Blakiston.

Transactions of the Medical Society of the State of Pennsylvania at its twenty-fourth Annual Session, June, 1873. Philadelphia: published by the Society.

Perityphilitis. By Wm. T. Bull, M. D. New York: D. Appleton & Co., 1873.

THE BUFFALO

**Medical and Surgical Journal**

PUBLISHED MONTHLY,

Containing Original Articles, Reports of Medical Societies and Hospitals, Editorial, Reviews, Correspondence, Army News, etc., etc; including the usual variety of Medical Periodical Publications. Specimen copies sent on application.

Address Buffalo Medical & Surgical Journal, Buffalo, N. Y.

TERMS OF SUBSCRIPTION, - - $3.00 PER YEAR, IN ADVANCE

